# Thorahcic SMARCA4-deficient undifferentiated tumors with ganglioneuroma and enchondroma: implications for SLC7A11 and ARID1A expression: a case report

**DOI:** 10.1186/s13000-022-01205-8

**Published:** 2022-02-12

**Authors:** Yusuke Kito, Keisuke Kawashima, Chiemi Saigo, Masayoshi Hasegawa, Shusuke Nomura, Takuya Mikamo, Yuki Hanamatsu, Yasuhiro Matsuo, Tamostu Takeuchi

**Affiliations:** 1 Department of Pathology and Translational Research, Gifu University Graduate School of Medicine, Yanagido 1-1, Gifu, 501-1194 Japan; 2 Department of Diagnostic Pathology, Matsunami General Hospital, 185-1, Dendai Kasamatsu-cho, Hashima-gun, Gifu, 501-6062 Japan; 3 Department of Respiratory Medicine, Matsunami General Hospital, 185-1, Dendai Kasamatsu-cho, Hashima-gun, Gifu, 501-6062 Japan

**Keywords:** SMARCA4-deficient thoracic sarcoma, SMARCA4-deficient non-small cell lung carcinoma, Thoracic SMARCA4-deficient undifferentiated tumors, ARID1A, SLC7A11, Ganglioneuroma, Enchondroma, Incomplete Carney complex

## Abstract

**Background:**

SWI/SNF-related, matrix-associated, actin-dependent regulator of chromatin, subfamily A, member 4-deficient thoracic sarcoma (SMARCA4-DTS) is a rare disease that has recently been described as an entity. It is characterized by an aggressive clinical course and specific genetic alterations. As an immunohistological feature, the tumors are deficient in SMARCA4 and SMARCA2 and express sex-determining region Y (SRY)-box 2 (SOX2). Occasionally, there are cases that are less frequent and difficult to distinguish from SMARCA4-deficient non-small cell lung carcinoma (SMARCA4-dNSCLC). Therefore, the 5th edition of the World Health Organization (WHO) classification describes thoracic SMARCA 4-deficient undifferentiated tumors (SMARCA4-UT). In contrast, Carney’s triad is a syndrome that combines three rare soft tissue tumors: gastric leiomyosarcoma, pulmonary chondroma, and extra-adrenal paraganglioma. Protein kinase cAMP-dependent type I regulatory subunit alpha (PRKAR1A) has been proposed as the causative gene. Both diseases are valuable cases; moreover, there have been no previous reports of their coexistence.

**Case presentation:**

A 43-year-old man visited our hospital because of respiratory distress. Computed tomography revealed a large mass measuring 55 mm in the upper lobe of the right lung and front mediastinum, with metastases in the surrounding lymph nodes. Needle biopsy was performed for diagnosis, and histological examination of the samples revealed monotonous epithelioid-like cells with loose binding and sheet-form proliferation. The tumor cells had distinct nuclei with some rhabdoid-like cells. Immunohistochemical analysis revealed that the tumor cells were positive for AE1AE3, SOX2, CD34, and p53 and negative for SMARCA4 and SMARCA2. The patient died 6 months after admission, without any treatment. Autopsy revealed ganglioneuroma and enchondroma suggestive of an incomplete Carney complex.

**Conclusion:**

SMARCA4-UT is a rare and recently established disease. While it is difficult to diagnose, it is necessary to distinguish undifferentiated carcinoma, large cell carcinoma, Ewing sarcoma, and epithelioid sarcoma when diagnosing tumors involving the mediastinum. Moreover, cases of SMARCA4-UT with ganglioneuroma and enchondroma are very rare. We discuss and report a case of SMARCA4-UT in which we also examined ARID1A and SLC7A11expression.

## Background

SWI/SNF-related, matrix-associated, actin-dependent regulator of chromatin, subfamily A, member 4-deficient thoracic sarcoma (SMARCA4-DTS) is a newly defined disease entity with characteristic clinical, pathological, and molecular features [1]. This tumor is caused by an abnormality in SMARCA4/BRG1, which is involved in chromatin remodeling [2–4]. This tumor has been reported in various organs, including small cell carcinoma of the ovary, medulloblastoma, lung cancer, intrathoracic sarcoma, and sinonasal undifferentiated carcinoma [5]. Its clinical feature is its average age at onset, i.e., patients in their 40s–50s, most often in men and smokers. SMARCA4-DTS is refractory to treatment, progresses rapidly, and has poor prognosis. Its morphological features include monotonous epithelioid-like cells that proliferate in sheet-like form with loose connectivity [6]. The tumor cells have a large eosinophilic cytoplasm, appear as rhabdoid cells, and have large swollen nuclei and distinct nucleoli [7]. The immunohistological features include high positivity for SRY (sex-determining region Y)-box 2 (SOX2), CD34, Sal-like protein 4 (SALL4), and p53; keratin positivity in half of the reported cases; and general negativity for BRG1/SMARCA4, Claudin4, transcription termination factor 1 (TTF-1), desmin, and S100 calcium-binding protein P (S100P) [8]. SMARCA4-DTS is sometimes difficult to consistently distinguish from SMARCA4- deficient non-small cell lung carcinoma (SMARCA4-dNSCLC). Moreover, immunostaining of SMARCA4 disappears even in non-small cell lung cancer. Therefore, SMARCA4-DTS is difficult to diagnose, with differentiation points including the loose binding of tumor cells, formation of glandular cavities and papillary growth, and strong diffuse staining of pan-keratin [9]. In addition, such tumors were described in the 5th edition of the WHO classification as a disease concept called Thoracic SMARCA 4-deficient undifferentiated tumors (SMARCA4-UT). Therefore, we further investigated the pathology of this tumor by investigating how ARID1A, which is part of the same BAF complex as SMARCA4, changes in SAMRCA4 deficient tumors and examined the expression of SLC11A, a downstream factor of ARID1A.

In contrast, in 1977, Carney et al. reported a case in which two or more of the three lesions of gastric leiomyosarcoma, extra-adrenal ganglionoma, and pulmonary chondroma were observed as single disease groups, irrespective of whether they were metachronous or simultaneous [10]. In 1977, Carney also reported the clinical features of 79 cases of this syndrome with two or more signs, as it may take years for all three signs to appear [11]. These cases are considered to correspond to the incomplete form of Carney’s triad. While protein kinase A regulatory subunit 1-α (PRKAR1A) is the causative gene, the Carney Complex exhibits a variety of phenotypes and there are many symptoms for which the relationship with gene mutations is unknown [12].

## Case presentation

**Fig. 1:**
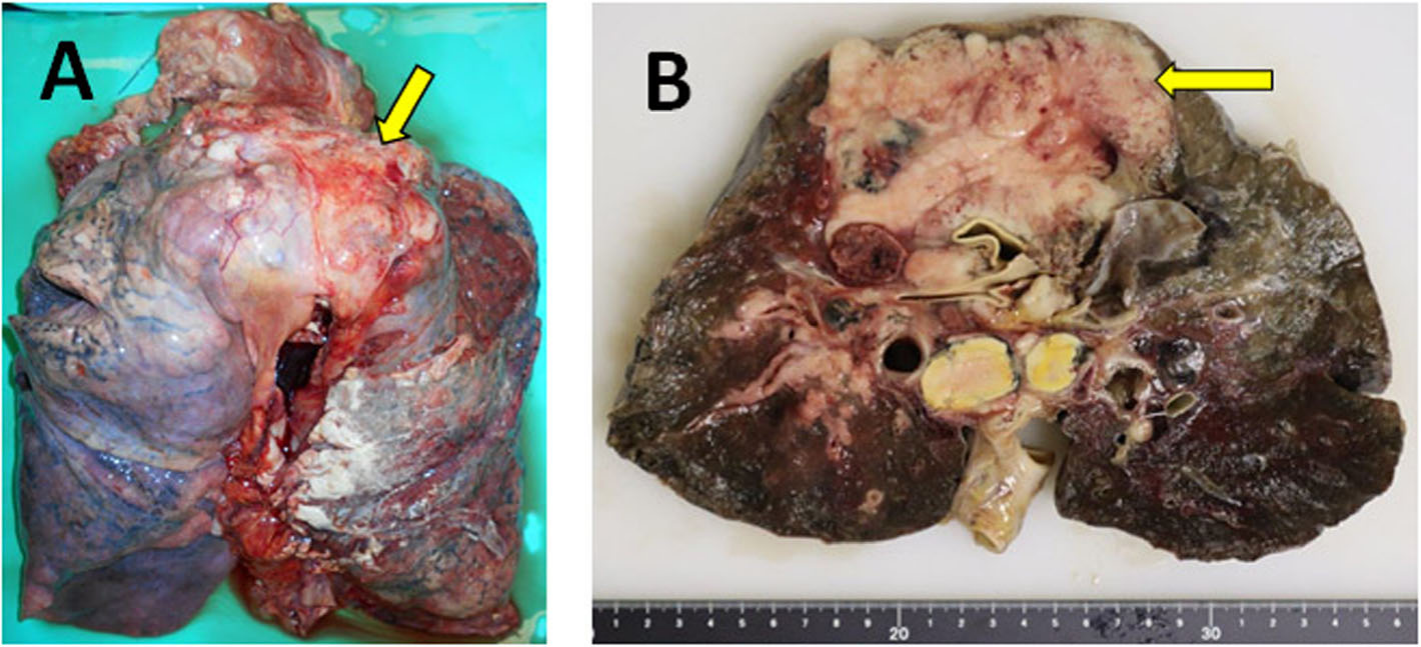
Representative macroscopic image of the case at the time of autopsy. **A** Lung and anterior mediastinal tumor at excision. The yellow arrow indicates the anterior mediastinal tumor. **B** Cross-section of the lung and mediastinal tumor. The yellow arrow indicates anterior mediastinal tumor

**Fig. 2:**
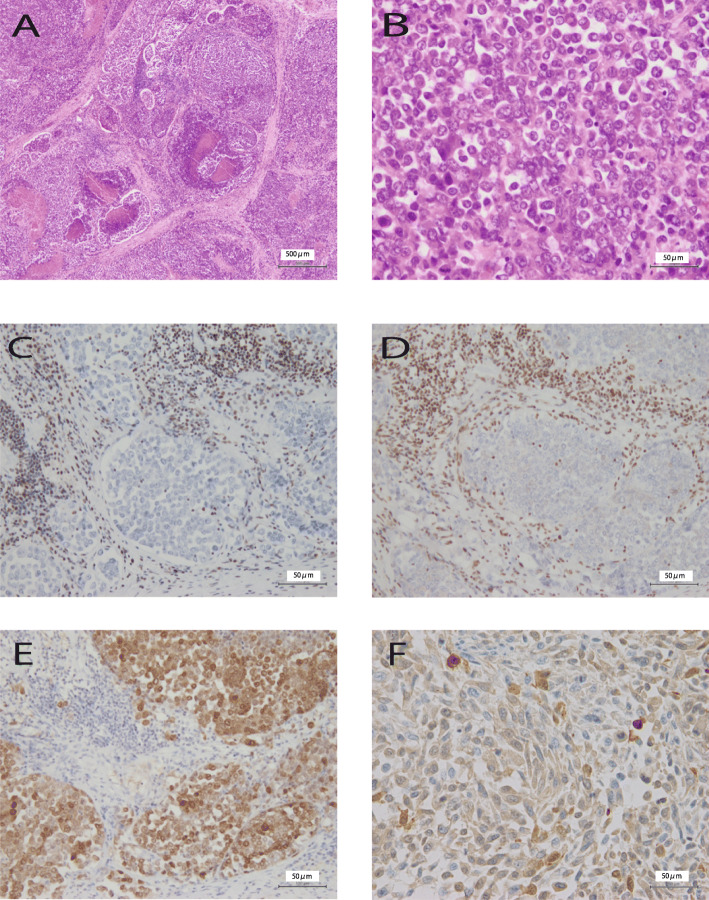
Representative histological findings and immunohistochemical features of SMARCA4-deficient carcinoma in the present case. **A** Necrotic and sheet-like growth pattern of monotonous epithelial-like cells. **B** The loose-binding tumor cells have distinct nucleoli. Rhabdoid-like cells are also observed. **C** Little or no immunoreactivity with anti-SMARCA4 antibodies. **D** Little or no immunoreactivity with anti-SMARCA2 antibodies. **E** Positive SOX2 immunoreactivity. **F** Weakly diffuse positive AE1/AE3 immunoreactivity

A 43-year-old man visited our hospital because of respiratory distress. He was a heavy smoker. Computed tomography revealed a large mass measuring 55 mm in the upper lobe of his right lung and front mediastinum, with multiple metastases observed in the surrounding lymph nodes. Lymph node biopsy was performed. The initial pathological diagnosis was NSCLC and poorly differentiated adenocarcinoma. While chemotherapy was administered, the tumor was refractory, and the patient died 6 months after admission. Subsequently, an autopsy was performed. Macroscopically, the tumor measured 10 cm in size and extended from the upper lobe of the right lung to the anterior mediastinum (Fig. 1A and B), with many lymph node metastases. The tumor was deeper than the submucosa and solid, opalescent, and necrotic. Histologically, the tumor showed a sheet-like growth pattern of monotonous epithelial-like cells. The tumor cells showed loose binding and distinct nucleoli, with some rhabdoid-like cells observed. Frequent mitotic figures and necrosis were also observed. Representative microscopic morphological features are shown in Fig. 2A and B. Immunohistochemical staining of paraffin-embedded tissues using a three-step ABC showed that the tumor cells were negative for desmin, S100P, TTF-1, p40, CK7, CK20, SMARCA4 (Fig. 2C), and SMARCA2 (Fig. 2D) and positive for SOX2 (Fig. 2E), CD34, p53, and AE1/AE3 (Fig. 2F).

**Fig. 3:**
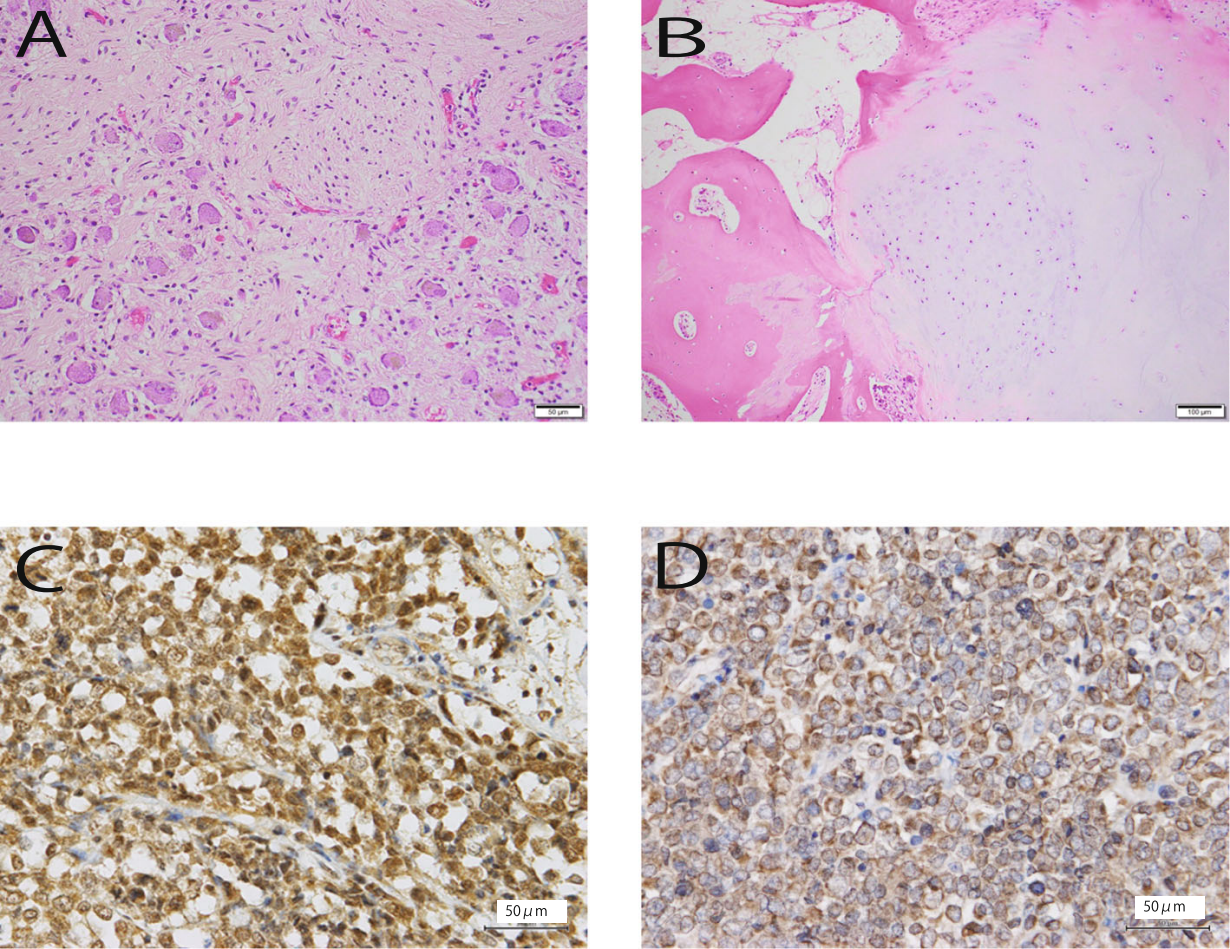
Representative histological findings. A ganglioneuroma in the adrenal gland (**A**) and an enchondroma in the left clavicle (**B**) are shown. **C** Immunoreactivity to SLC7A11, with positivity in lymphocyte and tumor cell nuclei. **D** Immunoreactivity to ARID1A, with positivity in lymphocyte nuclei, partial positivity in the nucleus of tumor cells, and positivity in the cytoplasm of tumor cells

While a partial ARID1A deficiency in the nuclei was observed (Fig. 3D), SLC7A11 expression was not decreased (Fig. 3C). The tumor cell cytoplasm was positive for ARID1A expression (Fig. 3D). Subsequent companion diagnostics for molecular therapies such as EGFR, ALK, and ROS-1 did not reveal mutations or fusion genes.

As a differential diagnosis, we considered lymphoma, NUT carcinoma, germ cell tumor, neuroendocrine carcinoma, large cell carcinoma, melanoma, and a variety of sarcomas such as rhabdoid tumors and epithelioid sarcoma. Lymphoma, NUT carcinoma, germ cell tumor, neuroendocrine carcinoma, and melanoma are unlikely to be detected by immunohistochemistry. The most difficult to distinguish are sarcomas, such as epithelioid sarcoma and SMARCA4-deficient NSCLC. In particular, the present case was diffusely positive for AE1/AE3; thus, SMARCA4-dNSCLC could not be ruled out. However, Yoshida et al. reported that half of the reported SMARCA4-DTS cases were pan-keratin-positive [13]. Moreover, Nambirajan et al. reported that SMARCA4-deficient NSCLC was CK7-positive and SOX2-negative, while SMARCA4-DTS was often CK7-negative and SOX2-positive [9]; however, the present case was CK7-negative and SOX2-positive. In addition, the age of onset is relatively young, and tumor development may occur in the mediastinum. Furthermore, no differentiation into adenocarcinoma or squamous cell carcinoma was confirmed in any tumor section. We diagnosed the present case as SMARCA4-DTS based primarily on the hematoxylin-eosin findings of loose binding and rhabdoid-like cells. According to the 5th edition of the WHO classification, these findings corresponded to thoracic SMARCA 4-deficient undifferentiated tumors. If frozen material is available, genetic analysis may reveal SMARCA4 deficiencies or mutations. Thus, this case may be a tumor, such as a borderline lesion between SMARCA4-DTS and SMARCA4-dNSCLC.

Autopsy revealed a ganglioneuroma in the adrenal gland (Fig. 3A) and an enchondroma in the left clavicle (Fig. 3B); in other words, two of the three signs of Carney’s triad. Therefore, the patient was diagnosed as having incomplete Carney’s triad. While PRKAR1A has been identified in recent years as one of the genes responsible for the Carney Complex, this complex exhibits a variety of phenotypes and there are many symptoms in which the relationship with gene mutations is unknown. Although Carney Complex was possible in this case, the patient had died, and no additional investigations, such as genetic screening, were performed. Therefore, a diagnosis of the Carney Complex could not be confirmed.

## Discussion and conclusions

SMARCA4-DTS is a group of diseases first reported by Le Loarer et al. in 2015 [1]. Their study investigated unclassified sarcoma by RNA sequencing and found that 19 cases showed inactivation of SMARCA4, which encodes the ATPase subunit of the BAF chromatin remodeling complex. SMARCA4-DTS has poor prognosis, with a median survival of 7 months [7]. Therefore, the development of effective treatments is urgently needed. A SMARCA4 mutant lung cancer cell line was recently reported to show oxidative phosphorylation (oxphos) activity and sensitivity to its inhibitor, IACS-010759 [13]. Thus, this drug may be an effective treatment for SMARCA4-DTS. In contrast, ARID1A was recently reported to promote the expression of SLC7A11 [14], which regulates the synthesis of the antioxidant glutathione. Moreover, ARID1A-deficient cancers have lower glutathione levels owing to reduced SLC7A11 expression. Therefore, research is underway on the treatment of ARID1A-deficient cancer by inhibiting glutathione and its synthesis [14]. Therefore, since BRG1 is another factor responsible for chromatin remodeling mechanism together with ARID1A, we hypothesized that SLC7A11 expression was decreased in SMARCA4- and ARID1A-deficient tumors. In short, SLC7A11 expression was reportedly decreased in ARID1A-deficient cancer [14]. Therefore, we investigated whether the expression of SLC7A11 was also decreased in tumors deficient in SMARCA4, a BAF complex, as synthetic lethal therapies are being developed for ARID1A-deficient cancers.

Subsequently, the expression levels of ARID1A and SLC11A were examined immunohistochemically. While a partial ARID1A deficiency was observed (Fig. 3D), SLC7A11 expression did not decrease (Fig. 3C). Thus, BRG1 deficiency was not directly related to SLC7A11 expression, while SLC7A11 expression was not affected by the presence or absence of ARID1A deficiency.

In contrast, ARID1A immunostaining revealed an interesting expression pattern (Fig. 3D). While ARID1A expression is usually observed in the cell nucleus, the present case, showed expression in the tumor cell cytoplasm (Fig. 3D). Previous reports of ARID1A immunostaining [15] [16] [17], have described nuclear expression patterns, which complete or partial defects observed in ovarian cancer, breast cancer, colon cancer, and cholangiocarcinoma.

There are no previous reports of the intracellular expression of ARID1A. Consistent with previous literature on ARID1A expression in other tumors, we also observed partial defects in ARID1A in the tumor in the present case. However, contrary to previous reports, we observed this expression in the cytoplasm. Tumor cells with cytoplasmic expression of ARID1A showed decreased nuclear expression of ARID1A, indicating a change in ARID1A localization from the nucleus to the cytoplasm. BRG1 deficiency did not significantly affect the SLC7A11 expression; however, BRG1 deficiency may have translocated the ARID1A protein from the nucleus to the cytoplasm. BRG1 deficiency may prevent ARID1A translocation to the nucleus or may prevent it from staying in the nucleus. The significance of cytoplasmic ARID1D expression remains unknown. Therefore, the causal relationship between this finding and the tumor etiology is unclear. Cytoplasmic ARID1A expression may be characteristic of SMARCA4A-UT, and changes in ARID1A expression patterns may help elucidate the functions of BRG1, ARID1A, and the BAF complex.

Ganglioneuroma and enchondroma were observed in the adrenal gland and the left clavicle, respectively. Carney’s triad is a rare multiple neoplastic association of pulmonary chondroma, gastrointestinal stromal tumor, and paraganglioma [10]. At least two tumors are required for its diagnosis [11]. Therefore, the findings in this case suggested an incomplete Carney triad. However, as the patient had died, no further assessments, such as genetic screening for PRKAR1A, were possible. Therefore, the diagnosis of Carney’s triad could not be confirmed. Ganglioneuroma and chondroma may be less associated with SMARCA4-UT but may be a subtype of the Carney complex.

In conclusion, we encountered a rare case of SMARCA4-UT. Further case reports may increase our understanding of the pathobiological and general properties of SMARCA4-deficient undifferentiated tumors. The present case was accompanied by a tumor suggestive of an incomplete form of Carney’s triad consisting of chondroma and ganglioneuroma. This was a very rare case in which two rare diseases were combined.

Interestingly, we detected ARID1A expression in cytoplasm of the tumor cells. No other tumors showing this expression pattern have been reported to date, although the relationship between this finding and tumor pathogenesis is unclear; thus, this case report is valuable.

## Data Availability

All data generated or analyzed during this study are included in this published article.

## Abbreviations

ARID1A: AT-rich interactive domain-containing protein 1A
BRG1: Brahma Related Gene 1
oxphos: oxidative phosphorylation
S100P: S100 calcium-binding protein P
SLC7A11: solute carrier family 7 member 11
SMARCA4: SWI/SNF-related, matrix-associated, actin-dependent regulator of chromatin, subfamily A, member 4
SMARCA4-DTS: SMARCA4-deficient thoracic sarcoma
SOX2: sex-determining region Y-box 2
SMARCA4-dNSCLC: SMARCA4-deficient non-small cell lung carcinoma
PRKAR1A: protein kinase A regulatory subunit 1-α
